# Contact-mediated intracellular delivery of hydrophobic drugs from polymeric nanoparticles

**DOI:** 10.1186/s12645-014-0008-4

**Published:** 2014-12-06

**Authors:** Sofie Snipstad, Sara Westrøm, Yrr Mørch, Mercy Afadzi, Andreas KO Åslund, Catharina de Lange Davies

**Affiliations:** Department of Physics, The Norwegian University of Science and Technology, Høgskoleringen 5, 7491 Trondheim, Norway; SINTEF Materials and Chemistry, Trondheim, Norway

**Keywords:** Contact-mediated, Drug delivery, Polymeric nanoparticles, Cellular uptake, Nile red

## Abstract

Encapsulation of drugs in nanoparticles can enhance the accumulation of drugs in tumours, reduce toxicity toward healthy tissue, and improve pharmacokinetics compared to administration of free drug. To achieve efficient delivery and release of drugs at the target site, mechanisms of interaction between the nanoparticles and cells and the mechanism of delivery of the encapsulated drug are crucial to understand. Our aim was to determine the mechanisms for cellular uptake of a fluorescent hydrophobic model drug from poly(butylcyanoacrylate) nanoparticles. Prostate adenocarcinoma cells were incubated with Nile Red-loaded nanoparticles or free Nile Red. Uptake and intracellular distribution were evaluated by flow cytometry and confocal laser scanning microscopy. The nanoparticles mediated a higher intracellular level and more rapid uptake of encapsulated Nile Red compared to model drug administered alone. The main mechanism for delivery was not by endocytosis of nanoparticles but by nanoparticle-cell contact-mediated transfer directly to the cytosol and, to a smaller extent, release of payload from nanoparticles into the medium followed by diffusion into cells. The payload thus avoids entering the endocytic pathway, evading lysosomal degradation and instead gains direct access to intracellular targets. The nanoparticles are promising tools for efficient intracellular delivery of hydrophobic anticancer drugs; therefore, they are clinically relevant for improved cancer therapy.

**Electronic supplementary material:**

The online version of this article (doi:10.1186/s12645-014-0008-4) contains supplementary material, which is available to authorized users.

## Background

Cancer treatment based on systemic chemotherapy is not cancer-specific, and toxic effects toward normal healthy tissue are challenging [1]. Nanoparticles carrying drugs may improve the tumour uptake of drugs and reduce their toxic effects through the enhanced permeability and retention (EPR) effect in tumour tissue [2]. EPR results in passive accumulation of nanoparticles in tumours due to the hyperpermeability of the vasculature and the lack of lymphatic drainage, whereas the nanoparticles are constrained to the blood vessels in normal tissue. To increase the fraction of nanoparticles reaching the tumour, a common strategy is to extend their time in systemic circulation [3]. This can be achieved by coating the nanoparticle surface with polyethylene glycol (PEG) [4,5] thereby preventing adsorption of opsonins via steric hindrance [6,7] and avoiding elimination through the mononuclear phagocyte system (MPS) [8,9]. Several different types of nanoparticles have been investigated as carriers for drug delivery [1,5,10], including polymeric nanoparticles [5,11]. Of these, poly(alkylcyanoacrylate) (PACA) nanoparticles are promising due to their ease and reproducibility of preparation, satisfying drug-loading capacity, low toxicity, and feasibility for scale-up production [12]. Some PACA nanoparticles are already in clinical development for cancer therapy [13]. We have developed a novel, multimodal, multifunctional drug delivery system consisting of microbubbles stabilised by polymeric PACA nanoparticles [14]. The nanoparticles can contain contrast agents for optical and magnetic resonance imaging as well as drugs and targeting ligands for combined diagnosis and therapy. The integration of nanoparticles and microbubbles into one single microparticle further makes them a promising agent for ultrasound-mediated delivery of encapsulated drugs to the tumour [15]. When developing nanoparticles for drug delivery, it is of crucial importance to understand the mechanism of interaction between the nanoparticles and cells and the mechanism of delivery of the encapsulated drug to achieve efficient delivery and release of drugs to the target. Interactions between cells and nanoparticles and the mechanisms for intracellular drug delivery have been investigated for various nanoparticles [16–21]. In most cases, endocytosis of the nanoparticles is the main mechanism for internalization [22,23], and subsequently the drug has to be released from the nanoparticle. Polymeric nanoparticles can employ various release mechanisms such as diffusion of the load, matrix swelling, polymer erosion, partition of the load, or a burst release effect depending on properties of the polymer and of the payload [24]. To be effective, drugs internalized by endocytosis of the nanoparticles depend on endosomal escape to reach the cytosol, to avoid lysosomal degradation [25,26]. Various possibilities for delivering substances directly into cytosol have been discussed [22,23,25]. Such directed cytoplasmic delivery into a target cell could provide an avenue for delivering greater amounts of agent with more efficient and immediate access to intracellular targets [27]. An example is delivery of load from nanoparticles to cells by collisional interactions [28,29].

The aim of the present work was to study the mechanisms of cellular uptake of the hydrophobic model drug Nile Red from poly(butylcyanoacrylate) (PBCA) nanoparticles *in vitro* and to determine whether the uptake was based on endocytosis of nanoparticles, extracellular release of Nile Red followed by diffusion into cells, or a contact-based transfer from nanoparticles to cells [30]. One of the advantages of Nile Red is its unique spectral properties: it emits fluorescence at different wavelengths depending on the hydrophobicity of the molecule binding to it [31]. Prostate cancer cells were incubated with nanoparticles encapsulating Nile Red or with free Nile Red dissolved in growth medium. The cellular uptake and intracellular distribution of Nile Red were studied using flow cytometry (FCM) and confocal laser scanning microscopy (CLSM).

## Methods

### Cell cultures

Human prostate adenocarcinoma cells (PC3, American Type Culture Collection) were grown in Dulbecco’s modified Eagle’s medium (DMEM, Gibco Invitrogen) supplemented with 10% foetal bovine serum (FBS, Sigma-Aldrich), and maintained in exponential phase at 37°C and 5% CO_2_.

### Synthesis and characterisation of nanoparticles

Solid biodegradable and biocompatible PBCA nanoparticles in water were synthesised in a single step by the miniemulsion process [14]. Briefly, oil-in-water emulsions were prepared by probe sonication (Branson Ultrasonifier, 3 min, 60% amplitude) of a monomer phase consisting of BCA (a kind gift from Henkel Loctite, 6 g) with co-stabiliser (hexadecane or Miglyol 810 N, 2% w/w) and the hydrophobic model drug Nile Red (Sigma-Aldrich, 0.03 or 0.15% w/w) in an acidic aqueous medium containing the surfactant sodium dodecyl sulfate (SDS, Merck, 12 mM, 24 ml, pH 1). Anionic polymerisation was carried out by adding a polyetheramine (Jeffamine M-1000, a kind gift from Huntsman Corporation, 0.05 M, 35 ml, pH 6), resulting in PEGylated nanoparticles. A dual-labelled nanoparticle containing 0.2% w/w Nile Red and 0.2% w/w p-HTAM (pentamer hydrogen thiophene acetic acid methyl ester, kindly provided by Linköping University) [32] was synthesized with Jeffamine M-2070 (kind gift from Huntsman Corporation, 0.05 M, 35 ml, pH 6) and the surfactant BrijL23 (Sigma-Aldrich, 19 mM, 30 ml). The two dyes are a good Förster resonance energy transfer (FRET) pair, and the particles were used to study uptake kinetics of different dyes. Excess PEG and surfactant were removed by dialysis against distilled water (6 shifts using a dialysis membrane with molecular weight cut-off of 12–14000). The size and the zeta potential of the nanoparticles (at pH 7) were measured by dynamic light scattering using a Zetasizer (Malvern Instruments). Successful PEGylation of the nanoparticle was verified by ^1^H-NMR [14]. The excitation and emission spectra for Nile Red in the nanoparticles were determined using a spectrophotometer (Olis RSM 1000).

### Incubation of cells with nanoparticles containing Nile Red or Nile Red in cell medium

Cells were incubated in growth medium supplemented with either nanoparticles containing Nile Red or free Nile Red in the same concentration range. The concentration of free Nile Red added to medium was estimated from the total amount of Nile Red added to the oil phase during particle synthesis. However, because SDS and PEG also associate with the oil droplets when nanoparticles are formed, the proportion of Nile Red in the final PEGylated particles will be smaller and is difficult to define exactly. The concentrations are estimated to be in the range of 4–6 ng/ml and 20–30 ng/ml for the particles with the lower and higher Nile Red content, respectively. A concentration of 20 μg/ml nanoparticles was used, corresponding to 10^4^ nanoparticles per cell. This concentration was chosen to avoid cytotoxicity, as concentrations above 20 μg/ml were found to be cytotoxic in the Alamar Blue assay (Additional file 1). To determine the number of nanoparticles per ml, a PBCA density of 1.1481 g/cm^2^ was used [33]. The nanoparticles with the lower Nile Red content were used in all experiments except for co-localisation studies between nanoparticles and early endosomes, in which particles with the higher Nile Red content were used to achieve similar fluorescence intensity as the labelled early endosomes.

For flow cytometric analysis, the appropriate number of cells was seeded in 6-well plates (Corning) to obtain 0.6 × 10^6^ cells in each well on the day of the experiment. Then the medium was replaced with nanoparticles or approximately 4 ng/ml free Nile Red. Eight ng/ml was used in one set of experiments to study the effect of a higher Nile Red concentration on uptake. Nile Red was also diluted in phosphate-buffered saline (PBS, Sigma-Aldrich), to determine whether proteins in the growth medium would affect the cellular Nile Red uptake. Cells were incubated for 15 min, 30 min, or 1, 2, 3, or 4 h to study the kinetics of uptake. The medium was removed, and the cells were rinsed 3 times with PBS before being detached. The effect of more extensive washing was also studied by centrifuging (Heraeus Megafuge 1.0R) at 1000 rpm for 3 min and resuspending the cells up to 3 times. In all experiments, the cells were placed on ice before flow cytometric analysis.

For microscopy, the appropriate number of cells were seeded in 8-well microscopy slides (Ibidi, Thistle Scientific) to obtain 30 000 cells in each well on the day of the experiment. The medium was replaced with medium containing either nanoparticles or free Nile Red, or the dual-labelled nanoparticles.

### Inhibition of endocytosis

To determine whether the uptake of Nile Red was due to endocytosis, cells were incubated with 10 μg/ml chlorpromazine or 70 μg/ml genistein (both from Sigma Aldrich), to inhibit clathrin-dependent and caveolae-mediated endocytosis, respectively [34]. The cells were pre-incubated at 37°C for 30 min with growth medium containing endocytosis inhibitors, before the nanoparticles were added for 3 h and the cells were washed and analysed by FCM. Incubation with encapsulated or free Nile Red were performed at 37°C or 4°C to investigate whether the cellular uptake of Nile Red was energy dependent.

### Labelling early endosomes and incubating with nanoparticles or Nile Red

Early endosomes were labelled using CellLight Early Endosomes-GFP (Invitrogen). 15 000 cells were seeded in 8-well microscopy slides and incubated for 24 h before replacing the medium with CellLight Early Endosomes-GFP at a concentration of 40 virus particles per cell. The cells were incubated for 24 h before replacing the medium with medium containing nanoparticles or 20 ng/ml free Nile Red. They were incubated for approximately 30 min before imaging with CLSM.

### Cellular uptake measured with flow cytometry

Cellular uptake of Nile Red or nanoparticles was analysed by FCM (Gallios, Beckman Coulter). A total of 10 000 cells were counted per sample. A 561 nm laser was used to excite Nile Red, and emitted fluorescence was detected at 620 nm using a 30 nm band pass filter. Cellular fragments and debris were excluded from the analysis by using a side-scatter versus forward-scatter histogram to establish a collection gate.

### Cellular uptake measured with confocal laser scanning microscopy

CLSM (Leica TCS SP5 or SP8) was used to study the intracellular distribution and uptake kinetics of Nile Red using a 63x/1.2 water objective. Live cell imaging up to 2,5 h was performed in 5% CO_2_ and 34-37°C using a 63x/1.4 oil objective. To excite Nile Red, a 561 nm laser was used on the SP5 and an argon laser at 488 nm or a white light laser enabling a tuneable excitation wavelength was used on the SP8.

The excitation and emission spectra of Nile Red depend on the hydrophobicity of the molecules the dye is binding to; this was used to distinguish between Nile Red bound to lipids, Nile Red in nanoparticles, or Nile Red associated with less hydrophobic molecules. An emisson-scan was captured, in which the emission spectrum of Nile Red was recorded for one excitation wavelength, and an excitation-emission-scan was captured, in which the detection range varied from 480 to 690 nm while the excitation wavelength varied from 470 to 670 nm using the white light laser.

The fluorochrome CellLight Early Endosomes-GFP was excited at 488 nm, and emission was detected at 500–530 nm. The dye p-HTAM was excited at 405 nm, and emission was detected at 470–530 nm. When studying FRET signal from the p-HTAM and Nile Red pair excitation of 405 nm was used, and emission was detected at 650–710 nm. The laser intensities and gains of the different detectors were adjusted with appropriate control samples to avoid cross-talk between the fluorochromes and to achieve a maximum signal with minimal saturation and background. The pinhole size was 1 airy unit. Fluorescence images, together with transmission microscopy images were captured.

### Release of Nile Red from nanoparticles

Because Nile Red is quenched in water, its release from nanoparticles into an aqueous solution could not be measured directly using a spectrophotometer. Therefore, cell medium containing 20 μg/ml nanoparticles was incubated at 37°C for 3 h before the suspension was centrifuged (Beckman Coulter Avanti J-30I) for 2 h at 21 000 rpm (~50 000 g). The supernatant from the centrifuged nanoparticle medium was added to cells, which were imaged after 15–30 min to determine whether Nile Red was released into the cell medium from the nanoparticles and taken up into cells.

Furthermore, cell medium with 20 μg/ml nanoparticles was incubated at 37°C for 3 h, centrifuged and Nile Red was extracted from the supernatant with hexadecane. The solutions were rotated overnight before standing still for several minutes to allow phase separation of hexadecane and the aqueous cell medium phase. The hexadecane phase was collected and analysed with a fluorescence spectrophotometer (Gemini XPS Fluorescence Microplate Reader, Molecular Devices) to determine the amount of released Nile Red.

To determine the total amount of Nile Red associated with the particles, the nanoparticles were dissolved in tetrahydrofuran (THF). The solutions were stirred for 4 h to dissolve the particles, and the amount of Nile Red was measured detecting the fluorescence spectroscopically after 24 h. Nanoparticles without Nile Red were dissolved and used as a control.

Excitation and emission maxima of 493 nm and 540 nm, respectively, were determined for Nile Red in hexadecane; similarly, maxima of 527 nm and 604 nm, respectively, were determined for Nile Red in THF. These maxima were used for the subsequent analysis. Hexadecane and THF with 0.001 to 0.1 μg/ml Nile Red were used to obtain standard curves.

### Data and regression analysis

FCM data were analysed with Kaluza Flow Cytometry Analysis software (Beckman Coulter) to determine the percentage of Nile Red-positive cells and the median fluorescence intensity (MFI), expressing the increase in fluorescence of the total population of cells relative to the autofluorescence.

Amira software (Visage Imaging) was used to construct 3-dimensional representations from z-stacks of images. The background fluorescence from the cytosol was removed by thresholding the CLSM co-localisation images and 3-dimensional representations.

Microsoft Excel was used for linear regression of standard curves. The kinetics of Nile Red uptake was analysed by non-linear regression using SigmaPlot. The exponential function f(t)= a(1 − e^−bt^) was fitted to the averages of the FCM data, where the coefficients a and b represent the maximum value and the rate constant, respectively. The goodness of each fit was determined by the square of the correlation coefficient (R^2^) and p-values <0.05 were considered statistically significant.

## Results

### Characterisation of nanoparticles

All three batches of nanoparticles had a hydrodynamic diameter of approximately 150 nm and a polydispersity index (PDI) of approximately 0.1, as measured by dynamic light scattering using the Zetasizer. The zeta potentials were −19 mV and −15 mV for the particles with the lower and higher Nile Red content, respectively, and −4 mV for the dual-labelled nanoparticles.

### Kinetics of the uptake of free or encapsulated Nile Red

**Figure 1:**
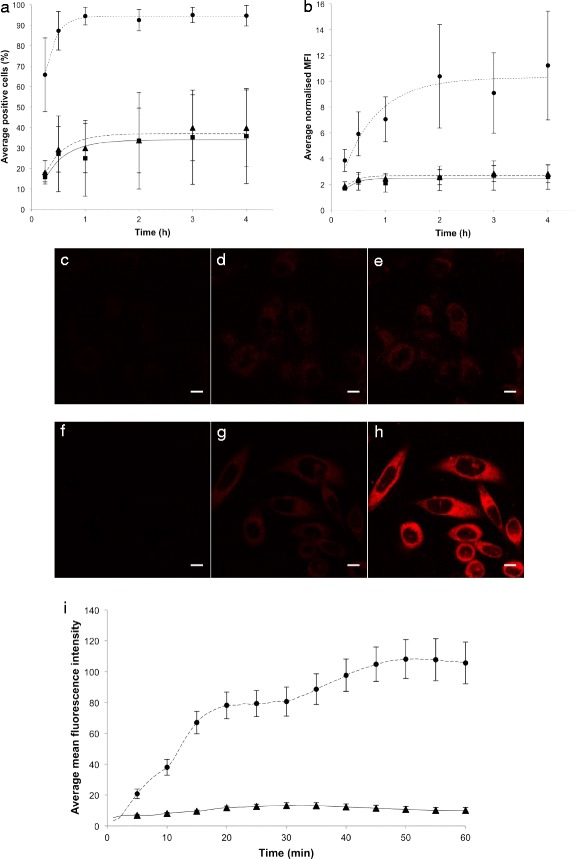
**Kinetics of cellular uptake of Nile Red encapsulated in nanoparticles (●) or dissolved in medium (▲) or PBS (■).** The cellular uptake was measured by FCM and is expressed as the percentage of Nile Red-positive cells **(a)** or median fluorescence intensity normalised to autofluorescence **(b)**. Each data point is the mean of 2–5 independent measurements. Bars indicate the standard deviation. The R^2^ values of the regression curves ranged from 0.73 to 0.99, and p-values were less than 0.014. CLSM images of cells incubated with Nile Red in medium **(c, d, e)** or Nile Red in nanoparticles **(f, g, h)** after 0 min **(c, f)**, 5 min **(d, g)**, and 60 min **(e, h)** of incubation. Scale bars are 10 μm. Nile Red was excited at 488 nm, and fluorescence was detected at 520–700 nm. CLSM images were recorded every min for 1 h. The average mean Nile Red fluorescence intensities from cells (n= 5-11) at each time point with standard deviation at every 5^th^ min are shown **(i)**. The mean intensity of each cell was determined by drawing regions of interest around each cell.

The uptake kinetics of encapsulated Nile Red and Nile Red dissolved in cell medium or PBS were compared, and the cellular uptake of Nile Red was higher when Nile Red was associated with nanoparticles (Figure 1). Nearly 100% of the cells had taken up Nile Red from nanoparticles compared to 37% when Nile Red was dissolved in medium (Figure 1a). The amount of Nile Red fluorescence per cell was approximately 4 times higher in cells incubated with nanoparticles compared to cells incubated with medium or PBS containing free Nile Red (Figure 1b). There was no significant difference in the cellular uptake of Nile Red dissolved in PBS or cell medium. The uptake kinetics fit well to a first order reaction both when measuring the percentage of Nile Red-positive cells and the average fluorescence intensity. The initial rate constant for the percentage of positive cells was approximately 2 times higher when the cells were incubated with nanoparticles compared to cells incubated with Nile Red in the medium (Table 1). Within 1 h, approximately all cells incubated with nanoparticles were fluorescent, and the amount of Nile Red internalised into these cells increased further and reached a maximum after approximately 2 h. Cells were also incubated with 8 ng/ml Nile Red dissolved in medium; in this case, the uptake was not twice as high but rather showed a maximum increase in MFI of 30%, indicating no linear relationship between concentration and uptake of free dye (Additional file 2).

**Table 1:** **Rate constants (b) and maximum values (a) from regression curves f(t)= a(1 − e** ^**−bt**^ **) fitted to data points from Nile Red uptake kinetics**

**Regression curve**	**Maximum value**	**Rate constant**
Nanoparticles, normalised MFI	MFI 10.3	1.5 /h
Free Nile Red in medium, normalised MFI	MFI 2.7	-*
Free Nile Red in PBS, normalised MFI	MFI 2.5	-*
Nanoparticles, percentage positive cells	94%	4.9 /h
Free Nile Red in medium, percentage positive cells	37%	2.6 /h
Free Nile Red in PBS, positive cells	34%	2.4 /h

Curves were fitted to the percentage of positive cells and to MFI after incubation with free Nile Red in medium, free Nile Red in PBS, or nanoparticle-associated Nile Red.

*Regression curves fitted to average fluorescence intensity of cells incubated with Nile Red in cell medium or PBS had only two data points describing the initial increase and were therefore not reliable.

The difference in cellular uptake of free and encapsulated Nile Red was confirmed by CLSM. Cells were imaged every minute up to 1 h, and images from 0-, 5-, and 60-min incubations are shown (Figure 1c-h). The uptake of encapsulated Nile Red was rapid, and CLSM images show that all the cells were positive for Nile Red after only a few minutes of incubation (Figure 1g, i). Intracellular fluorescence was observed throughout the entire cytosol, and the intensity was higher in the cells incubated with nanoparticles than in the cells incubated with Nile Red in the medium.

To exclude the possibility that rapid uptake could be cell line-specific, HeLa cells were incubated with nanoparticles and analysed by FCM. The uptake of Nile Red in HeLa cells after 15 min and 3 h of incubation was very similar to that in PC3 cells, both with respect to the percentage of positive cells and MFI (Additional file 3).

### Different uptake kinetics of Nile Red and p-HTAM

**Figure 2:**
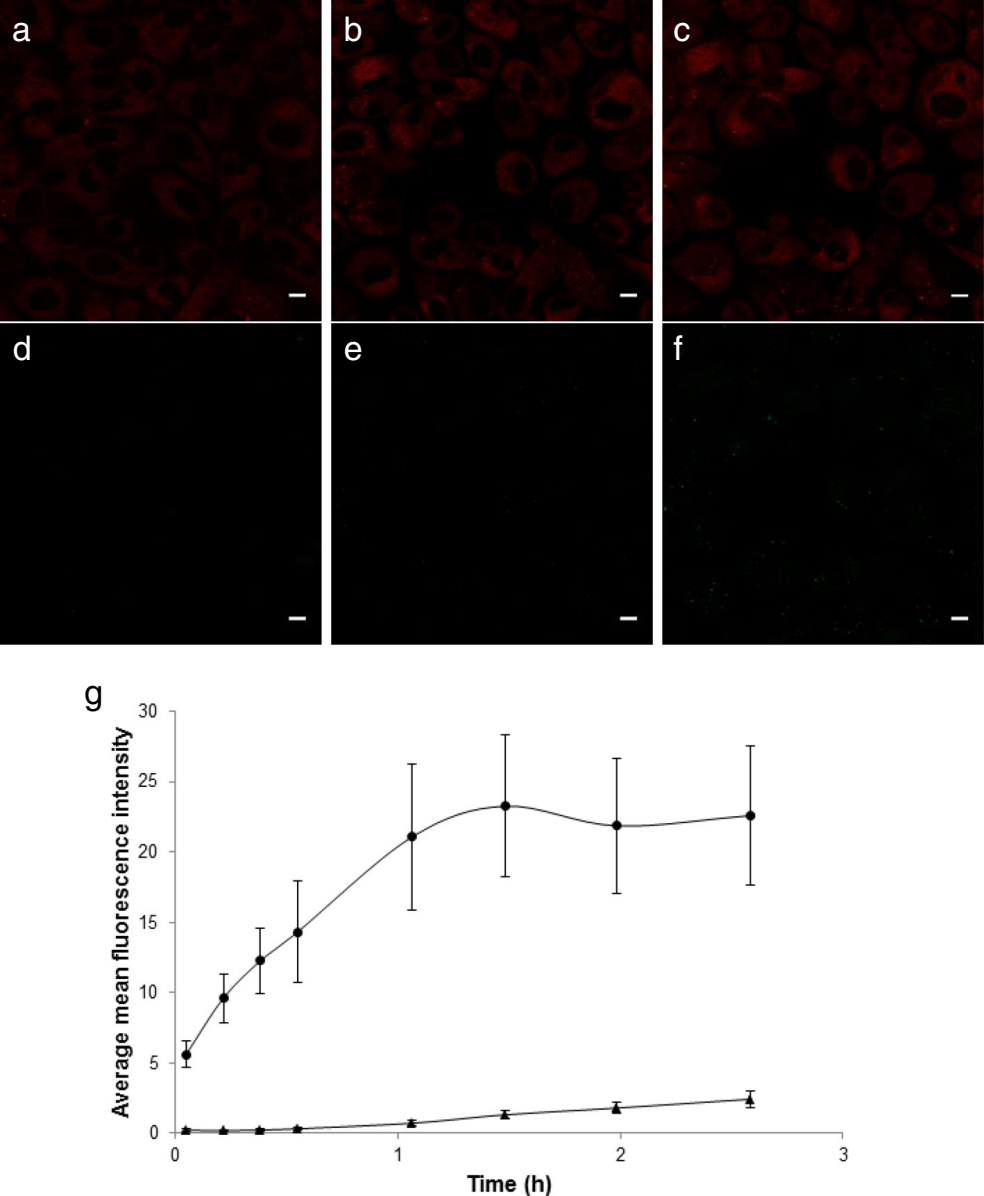
**CLSM images of cells incubated with dual-labelled nanoparticles containing Nile Red (red) (a, b, c) and p-HTAM (green) (d, e, f) after 8 min (a, d), 64 min (b, e) and 155 min (c, f) of incubation.** Scale bars are 10 um. Nile Red was excited at 540 and fluorescence detected at 650–710 nm, while p-HTAM was excited at 405 nm and detected at 470–530 nm. The average mean Nile Red (**●**) or p-HTAM (**▲**) fluorescence intensities with standard deviation from cells (n= 42-46) is shown from every 10 min for the first half hour, and from approximately every half hour after addition of particles **(g)**.

Dual-labelled nanoparticles were used to compare the uptake kinetics of different dyes encapsulated non-covalently in the same nanoparticle. Nile Red showed a rapid uptake and stained the entire cell after minutes of incubation. The uptake of p-HTAM was significantly slower, fluorescence was seen only from a few located spots in the cells after approximately half an hour and the number of spots increased with time (Figure 2). No FRET signal in the 650–710 nm channel was detected.

### Inhibition of endocytosis

**Figure 3:**
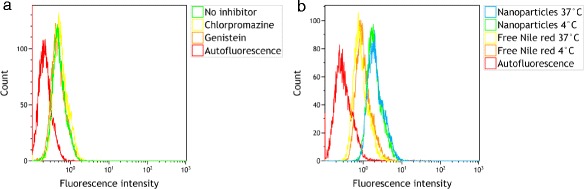
**Flow cytometry histograms illustrating cellular uptake and inhibition of uptake of Nile Red.** Cells incubated with nanoparticles for 3 h together with endocytosis inhibitors chlorpromazine or genistein **(a)** and cells incubated at 37°C or 4°C with either nanoparticles or free Nile Red for 1 h **(b)**.

To study whether endocytosis was responsible for the cellular uptake of Nile Red from nanoparticles, endocytosis was inhibited either by inhibitors or by incubation of the cells at 4°C. The inhibitors chlorpromazine and genistein had no effect on the Nile Red uptake (Figure 3a), indicating that the cellular uptake of Nile Red was not due to endocytosis of the nanoparticles. This was further confirmed by incubation with nanoparticles at 4°C. The cellular uptake of Nile Red after incubation for 1 h at 4°C and 37°C was similar, both for cells incubated with Nile Red in the medium and Nile Red associated with nanoparticles (Figure 3b). This demonstrates that the uptake of Nile Red is not an energy-dependent process, but rather is likely due to diffusion.

### Decreasing cellular Nile Red fluorescence by washing cells

**Figure 4:**
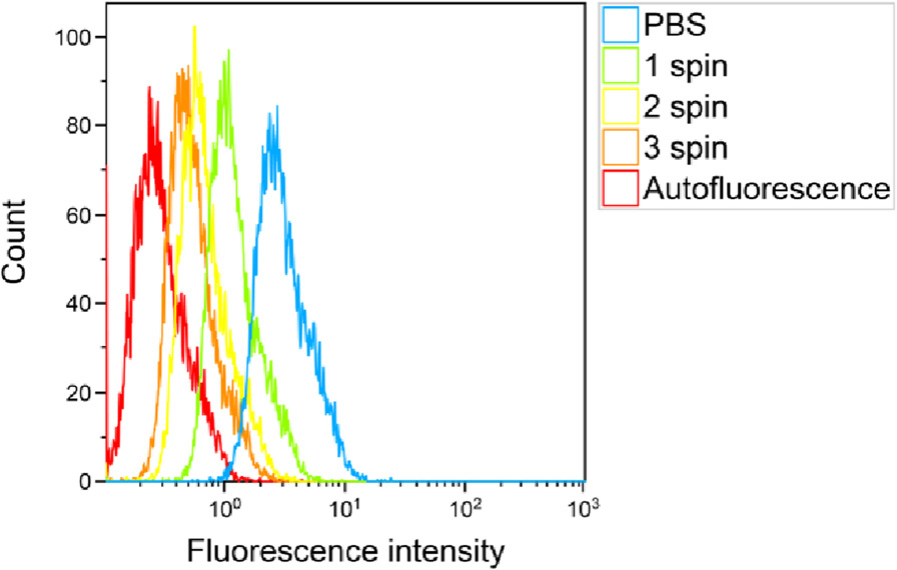
**Flow cytometry histograms illustrating the effect of washing on cellular Nile Red fluorescence after incubation with nanoparticles.** After incubation of 3 h at 37°C, the cells were rinsed 3 times with PBS and centrifuged and resuspended in medium 0 to 3 times.

To remove any nanoparticles or Nile Red binding to the cell surface, the adherent cells were rinsed 3 times with PBS. Furthermore, cells incubated with nanoparticles were washed by centrifugation and resuspended in medium containing serum up to 3 times. Nile Red fluorescence from cells incubated with nanoparticles for 3 h decreased with washing (Figure 4). After the third centrifugation, the median Nile Red fluorescence intensity was nearly reduced to the level of autofluorescence. The first centrifugation reduced the percentage of positive cells from 100% to 77%, the next to 27%, and the last to approximately 14%.

### Intracellular distribution of Nile Red

**Figure 5:**
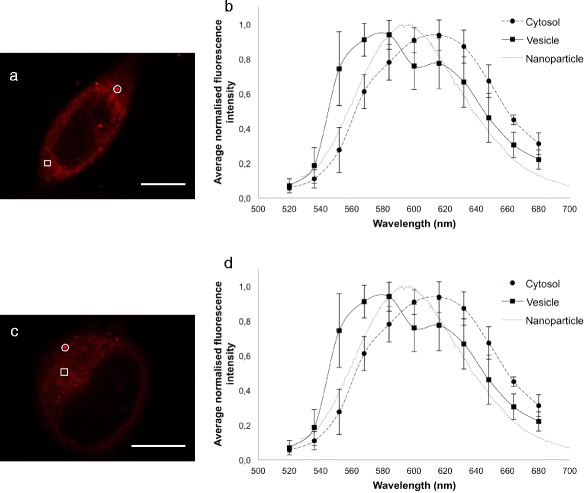
**Representative CLSM fluorescence images and emission spectra of cells incubated with nanoparticles containing Nile Red (a, b) or free Nile Red (c, d) for 1 h.** Scale bars are 10 μm. Nile Red was excited at 488 nm, and fluorescence was detected from 520 nm to 700 nm. Examples of regions of interest where emission spectra were captured from the cytosol (squares) and vesicular structures (circles) are shown. From an emission-scan with an excitation wavelength of 488 nm, the emission spectra from the cellular areas of interest are shown together with the emission spectrum from Nile Red in nanoparticles as measured by spectrophotometry **(b, d)**. In total, 8 cells incubated with nanoparticles were analysed with 12 regions of interest from the cytosol and 23 regions of interest from vesicles **(b)**. Three cells incubated with Nile Red were analysed with 6 regions of interest from the cytosol and 8 regions of interest from vesicles **(d)**.

Cells incubated with either Nile Red associated with nanoparticles or free Nile Red in medium showed similar intracellular distribution of Nile Red, although the fluorescence intensity was higher for cells incubated with nanoparticles. Nile Red was located in structures resembling vesicles, and it also showed diffuse cytosolic staining (Figure 5a, c). Spectral analysis of the fluorescence in vesicular structures and in the cytosol showed maximum emission at 584 nm and 616 nm, respectively (Figure 5b, d), demonstrating that the environment in the vesicular structure was more hydrophobic than the molecules Nile Red was binding to in the cytosol. The emission spectra were similar for cells incubated with nanoparticles and with free Nile Red. The emission spectrum from nanoparticles with encapsulated Nile Red measured by spectrophotometry has been included for comparison and showed a maximum emission at 597 nm. No spectra were found in intracellular vesicles that overlapped that of the nanoparticles.

**Figure 6:**
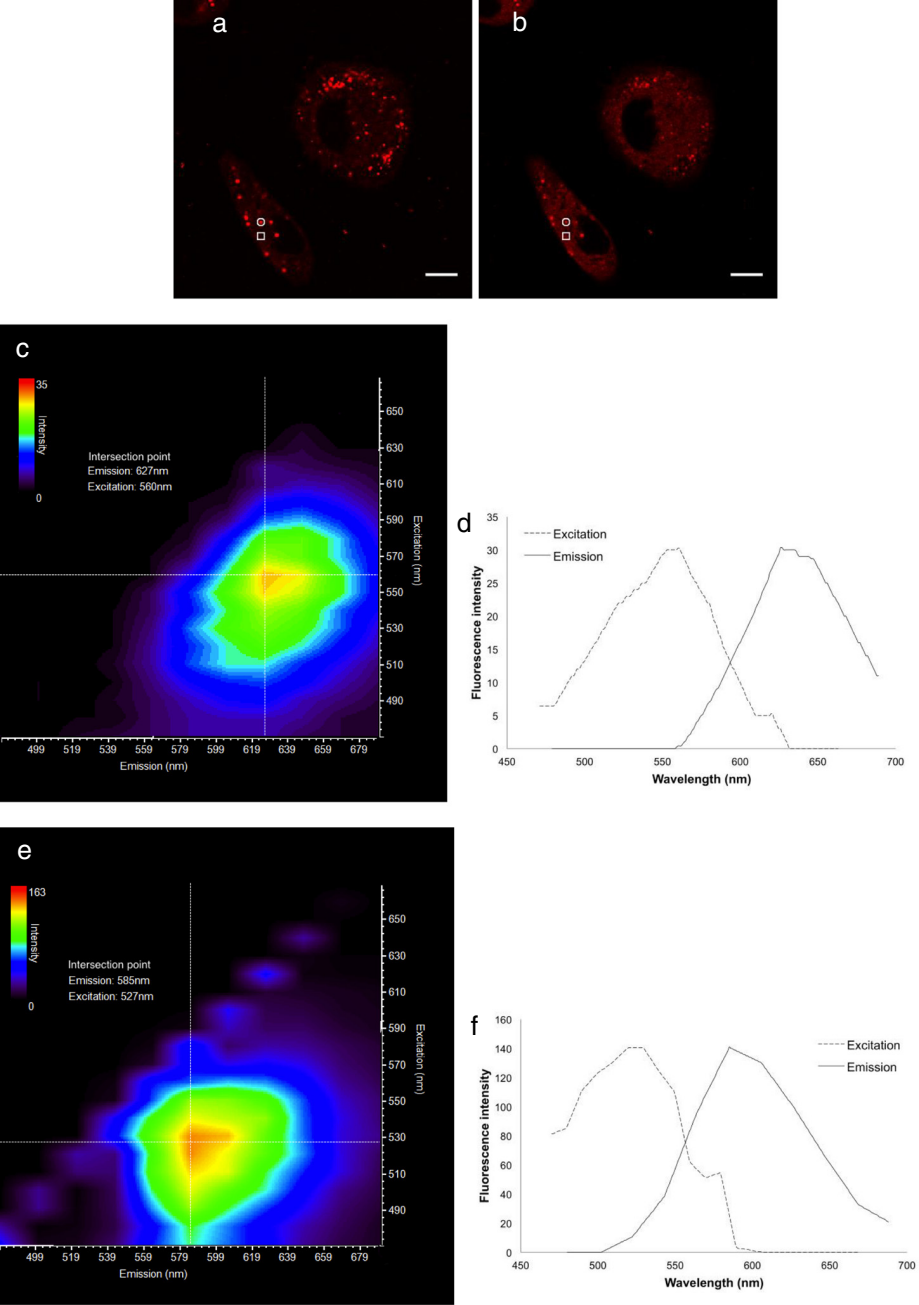
**CLSM fluorescence imaging and excitation-emission-scans of cells incubated with nanoparticles for 1 h.** Scale bars are 10 μm. Nile Red was excited at 488 nm and 542 nm with a white light laser, and fluorescence was detected at 550–590 nm **(a)** and at 650–720 nm **(b)**. An excitation-emission-scan was captured, and from the two regions of interest shown in the image, the excitation and emission intensity maps were obtained from the cytosol **(c)** and from a vesicular structure **(e)**. Corresponding excitation and emission spectra from the intensity maxima of the maps are shown in **(d)** and **(f)**, respectively.

To further exploit the spectral properties of Nile Red in the cytosol and vesicular structures, cells incubated with nanoparticles for 1 h were excited at 488 nm and 542 nm, and the fluorescence was detected at 550–590 nm and 650–720 nm, respectively (Figure 6a, b). From regions of interest representing a vesicular structure and cytosol, the excitation and emission intensity maps from the excitation-emission-scan along with the excitation and emission spectra from the intensity maxima of the maps were determined. The excitation and emission maxima of Nile Red in the cytosol were found to be 560 nm and 627 nm, respectively (Figure 6c, d); these maxima were 527 nm and 585 nm, respectively, in vesicular structures (Figure 6e, f).

**Figure 7:**
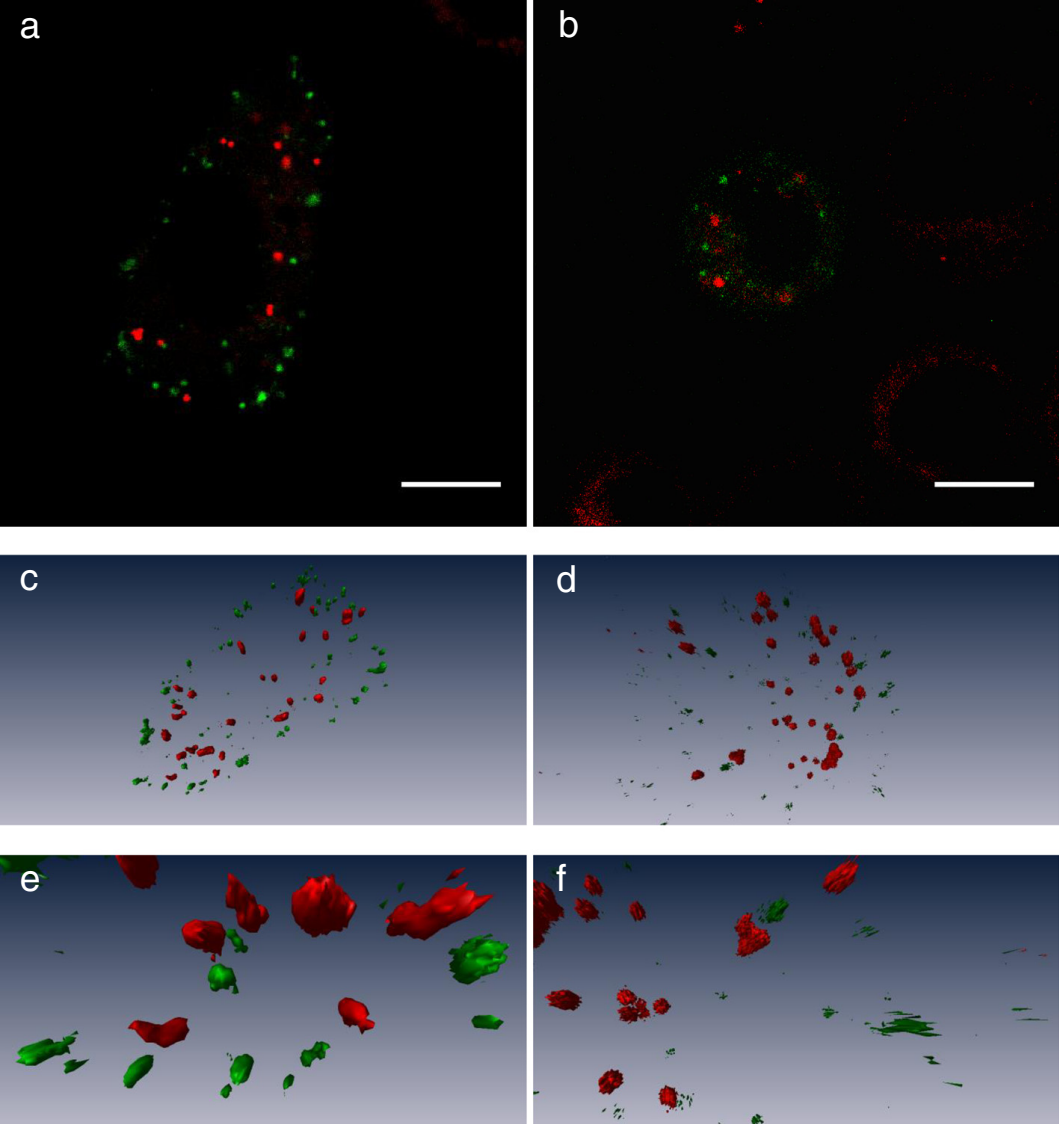
**CLSM images of early endosomes labelled in cells incubated for 30 min with nanoparticles with encapsulated Nile Red (a) or with free Nile Red in medium (b).** Below are 3-dimensional images generated from the CLSM z-stacks **(c and d)** together with zoomed in images of some vesicles **(e and f)**. Early endosomes are labelled with CellLight Early Endosomes-GFP, which is shown in green, Nile Red is shown in red. A 488 nm laser was used to excite CellLight GFP, and GFP fluorescence was detected at 500–530 nm, while Nile Red was excited at 540 nm with detection at 550–640 nm. Scale bars are 10 μm.

Cells with labelled early endosomes were incubated with Nile Red or nanoparticles to study co-localisation between Nile Red and early endosomes. From the CLSM images (Figure 7), we observed that there was very little or no co-localisation between Nile Red associated with vesicles (red) and early endosomes (green). The red and green spots were clearly separated from each other, and this was confirmed in the 3-dimensional z-stacks of the cells.

### Release of Nile Red from nanoparticles

**Figure 8:**
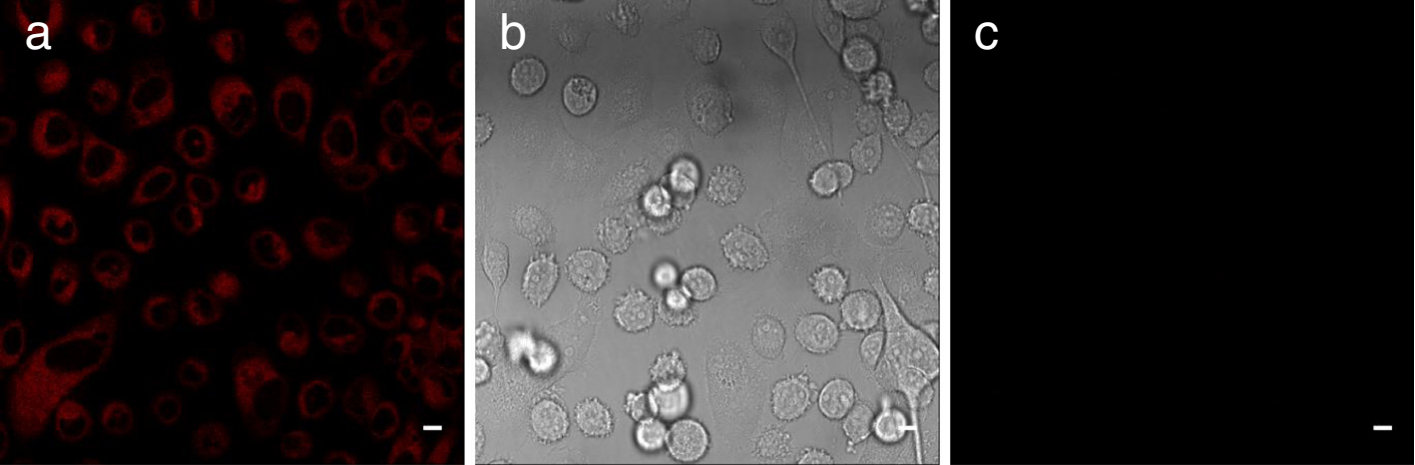
**CLSM images of cells incubated with supernatant from a centrifuged nanoparticle suspension.** Scale bars are 10 μm. Nile Red is shown in red. A fluorescence image obtained approximately 15 to 30 min after the addition of supernatant **(a)** and the corresponding transmission microscopy image **(b)** are shown together with a fluorescence image of untreated cells **(c)**. Nile Red was excited at 561 nm, and fluorescence was detected at 575–700 nm.

To determine whether Nile Red could be released from nanoparticles into the medium, a suspension of nanoparticles in cell medium was centrifuged, and the supernatant was added to cells before imaging with CLSM. It was found that Nile Red was released from the nanoparticles into the cell medium to some extent (Figure 8). From extraction experiments of Nile Red with hexadecane it was determined that approximately 50% of the total amount of Nile Red was released into the cell medium during 3 h (Table 2). The maximum release of Nile Red from nanoparticles was determined using THF which completely dissolves the particles. However, it cannot be determined to what extent the released Nile Red originated from surface-associated or encapsulated Nile Red. The values for total content of Nile Red in the nanoparticles were found to be in the expected range. Hence, the concentrations of 4 and 20 ng/ml that were chosen from the estimated Nile Red concentration range are in the same range as experimentally determined.

**Table 2:** **The amount of Nile Red released into the cell medium as a percentage of experimentally determined total Nile Red content in the nanoparticles**

**Release medium**	**Total content**	**Percent released**
2.9 ± 0.2 ng/ml	6.2 ng/ml	47%
12.7 ± 1.0 ng/ml	25.1 ng/ml	51%

“Release medium” is the measured amount of Nile Red released into the cell medium (n= 2) from the supernatant of centrifuged nanoparticle medium. The total amount of Nile Red in the particles was measured by dissolving the particles in THF.

## Discussion

The polymeric nanoparticles were found to increase both the intracellular level and uptake rate of the encapsulated model drug Nile Red compared to free Nile Red in growth medium or PBS. The delivery mechanism of encapsulated Nile Red was mainly by contact-mediated transfer directly into the cytosol and, to a smaller extent, release of Nile Red into the cell medium followed by diffusion into cells. Endocytosis of Nile Red encapsulated in nanoparticles was not observed.

There are several findings demonstrating that endocytosis was not responsible for the cellular uptake. Inhibition of clathrin-dependent and caveolae-mediated endocytosis did not result in decreased uptake of Nile Red. Furthermore, the energy-independent uptake at 4°C was similar to that at 37°C. Because all known endocytic pathways are energy-dependent [21], cooling the cells to 4°C will inhibit endocytosis [35,36]. The high uptake of Nile Red after incubation at 4°C thus suggests that diffusion was responsible for the uptake. The very rapid uptake also supports our hypothesis that diffusion is responsible, as endocytosis-dependent uptake would not be expected to occur only minutes after addition of nanoparticles, as was observed in CLSM. That Nile Red is not taken up by endocytosis was further confirmed by synthesizing a dual-labelled nanoparticle. The dye p-HTAM was chosen since we know from experience that it leaks out of nanoparticles at a slower rate than Nile Red and that it is a FRET-pair with Nile Red. If nanoparticles were taken up by endocytosis this would be seen by FRET. However, the fluorescence from Nile Red appeared within minutes, whereas the fluorescence from p-HTAM appeared after half an hour, and during the experiment no FRET was detected. Also, the more diffuse staining of Nile Red compared with localized p-HTAM staining indicates different uptake mechanisms.

Futhermore, Nile Red was removed from cells by extensive washing, indicating that intracellular Nile Red fluorescence did not originate from Nile Red inside nanoparticles, but rather from Nile Red binding to hydrophobic molecules in the cytosol. The reduction in Nile Red fluorescence can be explained by efflux of Nile Red from cells into the surrounding medium with serum, as reported in the literature [16,31]. Low molecular weight compounds can, once the concentration gradient outside the cell is removed, efflux the cell rapidly [21]. CLSM images showed no surface-bound dye, hence this can be ruled out as source of the fluorescence reduction.

Diffuse intracellular fluorescence from Nile Red was observed throughout the cytosol and Nile Red was located in spots resembling vesicles. The diffuse staining confirms that endocytosis is not responsible for the uptake [37]. Spectral analysis revealed that Nile Red in the vesicular spots was binding to more lipophilic molecules than the diffuse Nile Red staining. These vesicles were not early endosomes, as no co-localisation between Nile Red-stained vesicles and early endosomes was observed. Nile Red is sensitive to the degree of lipid hydrophobicity [31,38]; thus, lipid droplets or membranes with a high percentage of non-polar lipids are easily identified as yellow spots, while membranes with polar lipids are stained in the red spectral range [39–41]. Red fluorescence could also occur from Nile Red binding to proteins [31]. The similarity in emission spectra from vesicles and cytosol in cells incubated with nanoparticles and cells incubated with free Nile Red suggest that intracellular Nile Red is not associated with nanoparticles and that Nile Red taken up into cells has already dissociated from the particles in the extracellular environment. Furthermore, the excitation-emission-scan suggested that the intracellular Nile Red is likely bound to something else than nanoparticles, as the excitation and emission maxima do not correspond to those of Nile Red in nanoparticles, which has excitation and emission maxima of 540 and 600 nm, respectively. Thus, the spectral analysis indicates that all Nile Red was released from the nanoparticles before reaching the cytosol. The spectral analysis is consistent with the work of Greenspan *et al.*, showing that Nile Red fluorescence above 528 nm corresponded to small discrete spots in smooth muscle cells or lipids in macrophages, whereas the diffuse Nile Red fluorescence above 590 nm probably represents intracellular membranes and organelles [31].

Nile Red being released from nanoparticles into the medium was likely due to the presence of serum proteins with hydrophobic domains in the solution, which has also been reported for poly(lactic-co-glycolic acid)(PLGA) nanoparticles and nanoemulsions [16,42]. The release of Nile Red from the polymeric nanoparticles could be due to three mechanisms: 1) Nile Red loosely associated with the particle surface may be released; 2) Nile Red may diffuse through the polymer matrix and into solution; or 3) surface erosion or degradation of the nanoparticles may result in release of Nile Red. The degradation of PBCA nanoparticles in a medium at pH 7.4 is limited during the first 3 hours [43]; thus, degradation of particles in not likely the main cause of Nile Red release. It is possible that in addition to encapsulated Nile Red, some Nile Red is also located on the surface of the nanoparticles, which is not completely removed by dialysis [44]. Thus, surface-bound Nile Red release is hard to differentiate from Nile Red diffusing from the polymer matrix.

As the cellular uptake of Nile Red dissolved in medium or PBS was found to be low and slow, it is not likely that much of the dye that is taken up in cells was first released from nanoparticles to medium. The low uptake and slow uptake kinetics of Nile Red dissolved in medium or PBS compared to Nile Red delivered by nanoparticles were most likely due to Nile Red aggregating and binding to proteins. The hydrophobic nature of the dye will cause it to spontaneously minimise contact with water. The concentration-independent uptake of Nile Red dissolved in the medium also suggests that dye-aggregates in the medium are not able to diffuse into the cells. The higher uptake rate of Nile Red from nanoparticles compared to free Nile Red thus indicates that contact-mediated transfer is the main mechanism responsible for intracellular delivery.

Collisions between nanoparticles and cells have been reported to cause contact-mediated transfer of Nile Red directly into the cytosol. Xu *et al.* showed that such a transfer can happen for Nile Red in PLGA nanoparticles [16], and others have also observed contact-based transfer of Nile Red from lipid nanoparticles [45] and nanoemulsions [30]. It has also been shown that poly(isobutylcyanoacrylate) (PIBCA) nanoparticles can increase the intracellular content of the hydrophilic drug doxorubicin compared to free drug, without being taken up by cells through endocytosis [46]. However, to the best of our knowledge, contact-mediated delivery of hydrophobic drugs between PBCA nanoparticles and cells has not been previously reported.

In accordance with other reports [44,47], this study demonstrates the importance of distinguishing between encapsulated dye and released dye when studying intracellular uptake of nanoparticles and not necessarily interpreting intracellular fluorescence as uptake of nanoparticles.

The study further illustrates that direct delivery of a hydrophobic drug into the cytosol mediated by collisions and contact between the nanoparticle and plasma membrane is an efficient way to deliver drugs normally unavailable for uptake. It has been shown *in vivo* that contact-facilitated drug delivery can be very effective, allowing a great reduction of the needed dose [48], which will also lead to less toxic effects of drugs. Thus, the polymeric PBCA nanoparticle can be used for efficient delivery of hydrophobic drugs without the drugs entering the endocytic pathway, thereby avoiding lysosomal degradation. Even though the PBCA nanoparticles must be optimised to avoid premature release of their payload into the circulation and ensure efficient delivery to the target cells, they hold great promise for delivering hydrophobic drugs to tumour cells.

## Conclusion

Polymeric nanoparticles were found to mediate a higher intracellular level and a more rapid uptake of the encapsulated model drug compared to administration of the model drug alone; therefore, the nanoparticles could be used as a generic carrier of hydrophobic drugs for efficient drug delivery. The main mechanism of delivery was not via endocytosis of nanoparticles, but rather via nanoparticle-cell contact-mediated transfer directly to the cytosol and, likely to a smaller extent, release of payload from particles to the medium, followed by diffusion into cells. A contact-mediated mechanism of delivery into the cytosol could enable effective delivery of anticancer drugs directly to the intracellular molecular targets. The contact-based transfer mechanism and increased uptake of encapsulated drugs versus non-encapsulated drugs could also be exploited for the delivery of hydrophobic anticancer drugs to improve cancer therapy.

## Additional files

Additional file 1:
**Cytotoxicity of nanoparticles after 3 h exposure measured by the Alamar Blue assay (n= 2).** Cell viability is expressed as a percentage of control samples without nanoparticles, as a function of nanoparticle concentration. Additional file 2:
**PC3 cells incubated with 4 or 8 ng/ml free Nile red for 1 h at 37°C.** Cellular uptake measured by FCM. Additional file 3:
**PC3 and HeLa cells incubated with nanoparticles for 3 h at 37°C.** Cellular uptake measured by FCM.
