# Erratum to: Contact-mediated intracellular delivery of hydrophobic drugs from polymeric nanoparticles

**DOI:** 10.1186/s12645-015-0011-4

**Published:** 2015-07-29

**Authors:** Sofie Snipstad, Sara Westrøm, Yrr Mørch, Mercy Afadzi, Andreas K O Åslund, Catharina de Lange Davies

**Affiliations:** Department of Physics, The Norwegian University of Science and Technology, Høgskoleringen 5, 7491 Trondheim, Norway; SINTEF Materials and Chemistry, Trondheim, Norway

## Erratum to: Cancer Nanotechnology (2014) 5:8 DOI 10.1186/s12645-014-0008-4

After publication it was noted by the author that Figure 5 in the article ‘Contact-mediated intracellular delivery of hydrophobic drugs from polymeric nanoparticles’ (Snipstad et al. 2014) was published incorrectly. Panel b and panel d were identical. The corrected figure is shown below (Figure 1).

**Figure 1:**
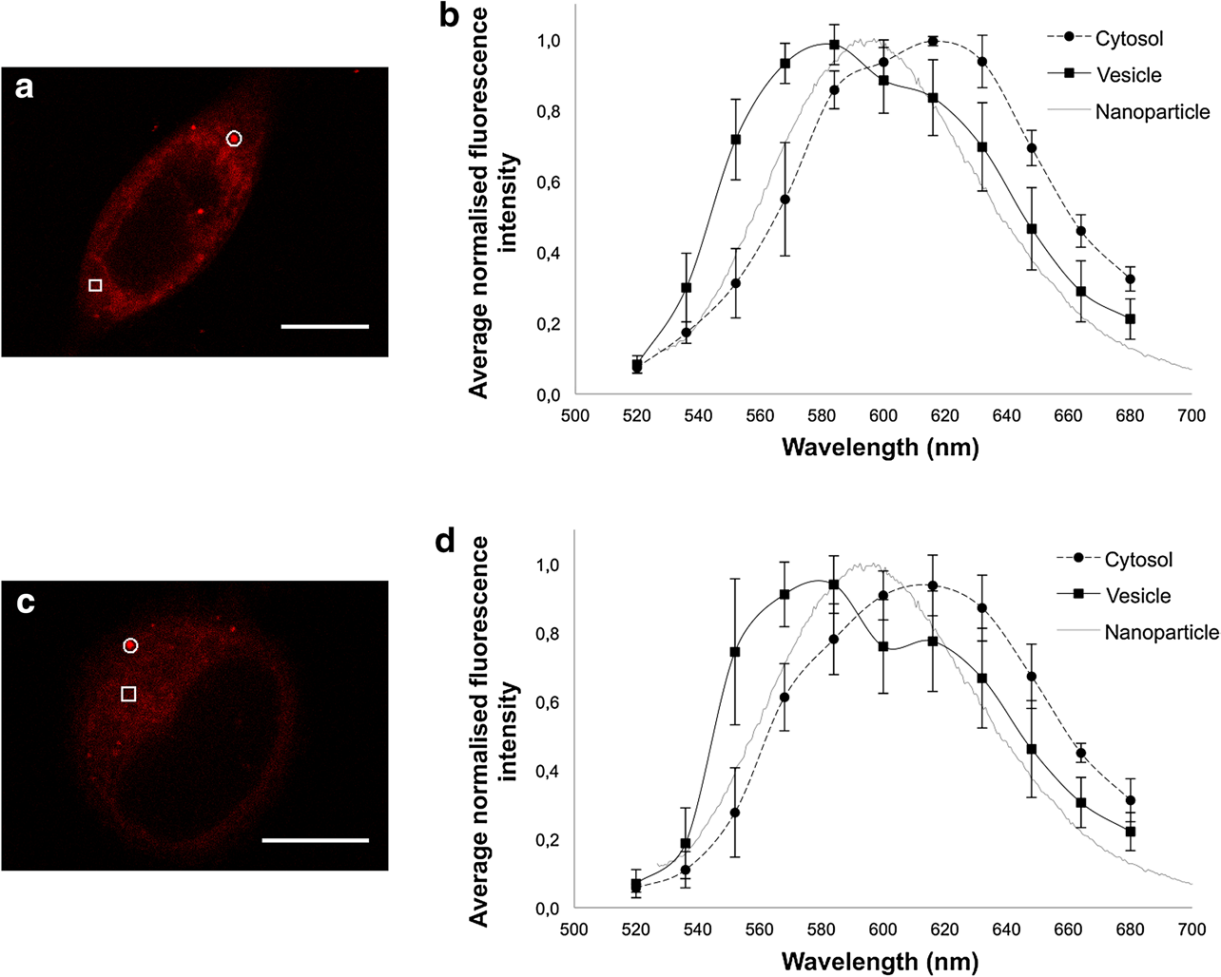
Representative CLSM fluorescence images and emission spectra of cells incubated with nanoparticles containing *Nile red* (**a**, **b**) or free *Nile red* (**c**, **d**) for 1 h. *Scale bars* are 10 μm. *Nile red* was excited at 488 nm, and fluorescence was detected from 520 to 700 nm. Examples of regions of interest where emission spectra were captured from the cytosol (*squares*) and vesicular structures (*circles*) are shown. From an emission-scan with an excitation wavelength of 488 nm, the emission spectra from the cellular areas of interest are shown together with the emission spectrum from *Nile Red* in nanoparticles as measured by spectrophotometry (**b**, **d**). In total, 8 cells incubated with nanoparticles were analysed with 12 regions of interest from the cytosol and 23 regions of interest from vesicles (**b**). Three cells incubated with *Nile Red* were analysed with 6 regions of interest from the cytosol and 8 regions of interest from vesicles (**d**).
