# Supplementary figures and images for: Contact-mediated intracellular delivery of hydrophobic drugs from polymeric nanoparticles
Source: Cancer Nanotechnol. 2014 Dec 6;5(1):8. doi: 10.1186/s12645-014-0008-4 (PMC4355425; doi:10.1186/s12645-014-0008-4)

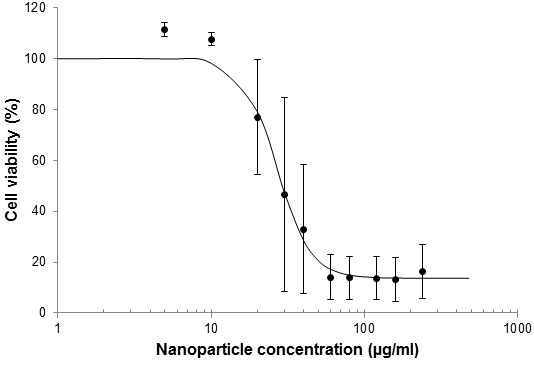


Additional file 1

Supplement: Additional file 1: — Cytotoxicity of nanoparticles after 3 h exposure measured by the Alamar Blue assay (n= 2). Cell viability is expressed as a percentage of control samples without nanoparticles, as a function of nanoparticle concentration. [file 12645_2014_8_MOESM1_ESM.docx]

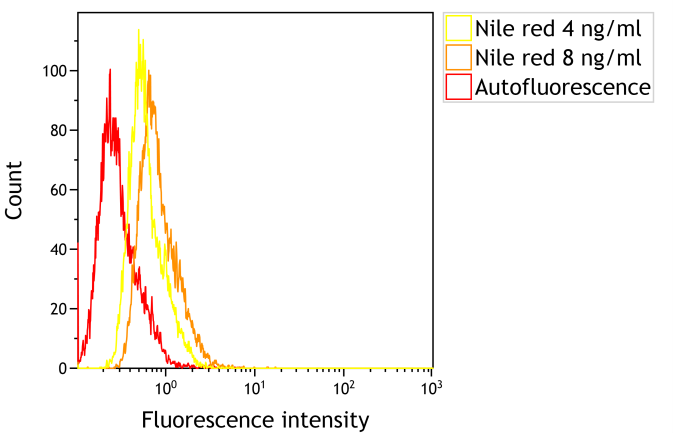


Additional file 2

Supplement: Additional file 2: — PC3 cells incubated with 4 or 8 ng/ml free Nile red for 1 h at 37°C. Cellular uptake measured by FCM. [file 12645_2014_8_MOESM2_ESM.docx]

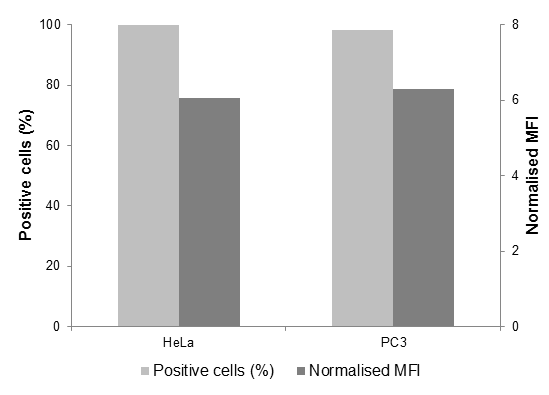


Additional file 3

Supplement: Additional file 3: — PC3 and HeLa cells incubated with nanoparticles for 3 h at 37°C. Cellular uptake measured by FCM. [file 12645_2014_8_MOESM3_ESM.docx]
